# Surfactant-assisted alkaline pretreatment and enzymatic hydrolysis of *Miscanthus sinensis* for enhancing sugar recovery with a reduced enzyme loading

**DOI:** 10.3389/fbioe.2022.918126

**Published:** 2022-07-22

**Authors:** Xiyu Cheng, Ying Luo, Yifan Gao, Shen Li, Chunming Xu, Shangyuan Tang, Yongkun Yang, Zehua Zhang, He Jiang, Hanli Xu, Shuobo Shi, Qiong Yan

**Affiliations:** ^1^ College of Life Sciences and Bioengineering, School of Physical Science and Engineering, Beijing Jiaotong University, Beijing, China; ^2^ Key Laboratory of Cleaner Production and Integrated Resource Utilization of China National Light Industry, Beijing Technology and Business University, Beijing, China; ^3^ Shangrao Municipal Ecological Environment Bureau, Shangrao, China; ^4^ Beijing Advanced Innovation Center for Soft Matter Science and Engineering, Beijing University of Chemical Technology, Beijing, China

**Keywords:** surfactant-assisted alkaline pretreatment, surfactant-assisted enzymatic hydrolysis, *Miscanthus sinensis*, poly (ethylene glycol) 2000, biomass

## Abstract

Surfactants play a vital role in the delignification and saccharification of lignocellulosic biomass. A strategy for coupling surfactant-assisted alkaline pretreatment (SAP) with surfactant-assisted enzymatic hydrolysis (SEH) has been proposed for improving sugar recovery from a potential energy crop, *Miscanthus sinensis*. Poly (ethylene glycol) 2000 (PEG 2000) was found to be more efficient in SAP than in other tested surfactants. Compositional and structural analysis revealed that the SAP process with 1% of PEG 2000 produced more efficient lignin removal and microstructure disruption of the pretreated sample, thus indicating much higher reducing sugar yields of 544.4–601.2 mg/g compared to the samples that were untreated or pretreated by alkali alone. Moreover, SEH with 1% Tween 80, which could block the lignin-enzyme interactions, produced a substantial reduction of 33.3% in the enzyme loading to achieve a higher sugar recovery from the SAP sample.

## Introduction

Bioenergy production from different organic wastes has gained increasing attention (Atelge et al., 2020; Cui et al., 2022). Lignocellulosic biomass, including agricultural wastes (e.g., corn stalk, cotton stalk and rice straw), forestry wastes, and energy crops, which are non-food substrates and renewable source with reduced net emission of CO_2_, has been widely studied as substrates for production of ethanol and biogas (Phitsuwan et al., 2016; Tang et al., 2019; Xu et al., 2021). Given the limited farmland resources in China, the planting of energy crops on available marginal land, which is estimated to be approximately 5.5 million ha, is considered among the most promising methods for producing biofuel feedstock (Ferraz et al., 2020; Rezania et al., 2020). *Miscanthus sinensis* (*M. sinensis*), which is a perennial grass, is cultivated throughout most areas in China and is considered as a potential energy crop. Its attractive merits include effective nutrient cycling, high genetic variation, easy propagation, and high yield (27–38 t/ha) (Fu et al., 2018). Therefore, *M. sinensis* was selected as a model energy crop to investigate its potential application in this study.

Bioconversion of lignocellulosic biomass remains limited due to its recalcitrant structure. A key step in bioconversion is pretreatment (Rezania et al., 2020; Wu et al., 2021). An ideal pretreatment technology aims not only to enhance the enzymatic hydrolysis of lignocellulosic biomass by disrupting microstructure, changing components, and improving accessibility, but also to minimize sugar loss and by-products’ formation to obtain a high sugar yield in the pretreatment step at a reduced cost. In the past decades, different strategies (e.g., organic solvent, acid, alkaline, and hot water pretreatments) have been developed to increase enzymatic hydrolysis and the subsequent fermentation of varied biomass wastes (Meng et al., 2015; Sahoo et al., 2018; Wang et al., 2018). Typical techniques, such as steam explosion and hot water pretreatments, are effective for improving the bioconversion of pretreated samples; however, they show a relatively high formation of by-products and energy consumption due to their severe operational conditions (Michelin et al., 2016). Although other strategies, such as biological pretreatments, have a lesser energy requirement, they are time-consuming and/or can consume the fermentable sugars of raw materials due to microorganisms used in these pretreatments (Atelge et al., 2020; Xu et al., 2021). Until now, an ideal candidate that could well satisfy all the requirements for the ideal pretreatment process is yet to be discovered.

Lignin is an important obstacle to the efficient saccharification of lignocellulosic biomass since it impedes its availability to enzymes and microorganisms (Qing et al., 2010; Martin et al., 2022). Alkaline pretreatment, which produces an effective delignification and chemical swelling of fibrous cellulose, is considered among the most streamlined and efficient techniques (Li et al., 2016a, b; Shimizu et al., 2018). Enhanced enzymatic saccharification of various organic wastes such as bamboo, *Eucalyptus* samples, and pine foliage, was obtained by alkaline pretreatment (Li et al., 2016a, b; Pandey and Negi, 2015). However, it should be noted that the dissolved lignin, which forms hydrophobic compounds in the pretreatment liquid, could cyclically precipitate back on the surface of the pretreated biomass substrates (Maurya et al., 2013). As a result, the availability of cellulose and hemicellulose for enzymatic access will be impeded. Surfactants may decrease the surface tension between liquid phases in the pretreatment stage and extract these dissolved lignin compounds by the formation of an emulsion. Few studies have demonstrated that the addition of surfactants could decrease the redeposit of lignin on the cell wall surface of biomass, thereby further enhancing the efficiency of the enzymatic hydrolysis of biomass wastes, such as corn stalk, sugarcane tops, and pine fallen foliage (Kataria et al., 2018; Sindhu et al., 2018; Wang et al., 2020). Diverse surfactants (e.g., PEGs with different molecular weights) possess different hydrophilic and hydrophobic properties, which are of great importance for the improvement of the process performance. However, studies on the development of alkaline pretreatment and the subsequent enzymatic hydrolysis of energy crops assisted by different PEGs and other surfactants remain limited, and the underlying mechanism remains unclear.

In the present work, the impact of surfactants on alkaline pretreatment and enzymatic hydrolysis of *M. sinensis* was investigated for the development of a promising strategy by improving hydrolysis efficiency and/or reducing the amount of enzyme needed to achieve a given conversion. First, different surfactants, including PEG and Tween species, were screened to identify a suitable candidate for the development of an effective surfactant-assisted alkaline pretreatment (SAP). The composition and microstructure in response to the pretreatments were then investigated to better illustrate the exact roles of the pretreatment in biomass recalcitrance changing and subsequent improvement of the enzymatic hydrolysis. Moreover, considering the high enzyme cost of bioethanol production (Zhang et al., 2020; Xu et al., 2021), a surfactant-assisted enzymatic hydrolysis (SEH) process was developed, and coupled with SAP to explore the possibility of further improving the bioconversion efficiency of SAP sample with a reduced enzyme loading.

## Materials and methods

### Materials

Stalk samples of *M. sinensis* were collected from the Fujian province, China. These sun-dried samples were dried in an electronic oven at 60°C for more than 24 h to a constant weight. Afterward, milling of the samples was done using a plant miller, followed by sifting through a 20-mesh sieve.

### Pretreatment of stalk samples

Alkaline pretreatment (AP) and surfactant-assisted alkaline pretreatment (SAP) were performed in glass bottles in an autoclave. In brief, the dried samples were added to glass bottles containing 0–2.0% (w/v) surfactants (i.e., PEG, Tween, and Cetyltrimethylammonium bromide (CTAB)), and 0.6–1.0% (w/v) NaOH solutions, respectively, based on a solid loading rate of 10% (Li L. C. et al., 2020; Xu et al., 2021). Different surfactants, including PEG 400, PEG 2000, PEG 4000, PEG 6000, PEG 20000, Tween 60, and CTAB, were used in SAP. For all pretreatments, the samples were autoclaved at 121°C for 10 min. The pretreated stalk samples were cooled to room temperature and centrifuged. The supernatants were collected and stored at –20°C for further analysis. The solid residues were washed with deionized water until the obtained filtrates were neutral. The solid residues were then dried in an oven at 105°C to a constant weight. The dried solids were sealed in plastic bags and stored in a desiccator at room temperature until the following analysis or enzymatic hydrolysis.

### Enzymatic hydrolysis of stalk samples

The enzymatic hydrolysis of the raw and pretreated samples was conducted in a 250-ml conical flask using 50 mM of sodium acetate buffer (pH 5) containing 40 μl of tetracycline hydrochloride (25 mg/ml) (Tang et al., 2019). The raw and pretreated stalk samples were added based on a 2.5% solid loading and enzymatic hydrolysis was then performed at 50°C and 150-rpm shaking speed for 72 h. The cellulase obtained from Hunan Youtell Biochemical Co., Ltd (Hunan province, China) was used for the above enzymatic hydrolysis and the loading ratio of the cellulase was 15 filter paper unit (FPU)/g of the stalk substrates. One FPU is defined as the amount of the enzyme that produces glucose from filter paper substrates at 1 μmol/min in the above reaction mixtures at 50°C and pH 5. Reducing sugars in the enzymatic hydrolysate were measured by the standard method of the 3, 5-dinitrosalicylic acid (DNS) assay (Miller, 1959). For the surfactant-assisted enzymatic hydrolysis (SEH) of SAP samples, extra surfactants (i.e., 1% of Tween 80, Tween 20, or PEG 2000) were added with different enzyme loading ratios (8, 10, 12, or 15 FPU/g).

### Scanning electron microscopy (SEM) observation

The microstructure of the raw and pretreated stalk samples of *M. sinensis* was observed by SEM (Tang et al., 2019). The dried samples of *M. sinensis* were fixed in a specimen holder with aluminum tape. The surface of the samples was then sputtered by gold using a JEOL JEC-1200 sputter-coater (Tokyo, Japan). The specimens were then examined with a JEOL JSM-5600 LV scanning electron microscope (Tokyo, Japan) under a high vacuum. An accelerating voltage of 5.0 kV was used for the observations.

### Analysis

The sample mixtures were centrifuged after AP and SAP. Solid yields were calculated based on the residual total solid of the stalk samples after all pretreatments. The contents of cellulose, hemicellulose, and lignin were measured gravimetrically following the standard method of Goering and Van-Soest (Goering and Van-Soest, 1970; Xu et al., 2021). In brief, the neutral detergent fiber (NDF) level was determined gravimetrically by extracting the solid residue of different pretreatments with a neutral detergent (ND). The acid detergent fiber (ADF) level was then determined gravimetrically by extracting the solid residue of the ND extraction with an acid detergent (AD). Lignin content was then measured gravimetrically from the free ash after the solid residue of the AD extraction was extracted using sulfuric acid solution (72%). The cellulose content was obtained by subtracting the pre-ash lignin level from the ADF level. The ADF level was subtracted from the NDF level to obtain the hemicellulose content. The ash content of the solid residue was measured gravimetrically in a muffle furnace at 550°C for over 6 h. The contents of cellulose, hemicellulose, and lignin were calculated based on residual total solid. All experiments were performed in triplicates.

The determination of cellulose accessibility of untreated and pretreated stalks was done according to the direct red dye (DR28) adsorption method (Wiman et al., 2012). In brief, the stalk biomass (1%, w/v) was immersed in sodium citrate buffer (pH 4.8) with various dye concentrations (0–4 g/L). The mixtures were placed in the condition of 50°C and 150 rpm in a shaking incubator for 24 h. Centrifugation was used to collect the supernatant to determine the free dye content. The absorbed dye content difference before and after the adsorption was used to calculate the cellulose accessibility.

## Results and Discussion

### Screening of surfactants for SAP

**FIGURE 1:**
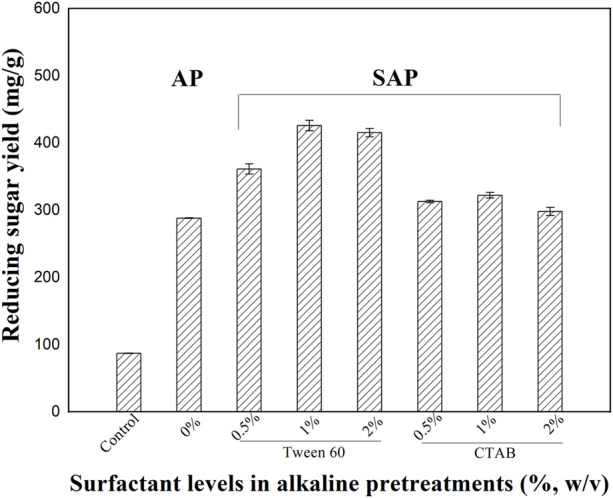
The effect of SAP with Tween 60 and CTAB surfactants on enzymatic hydrolysis.

**FIGURE 2:**
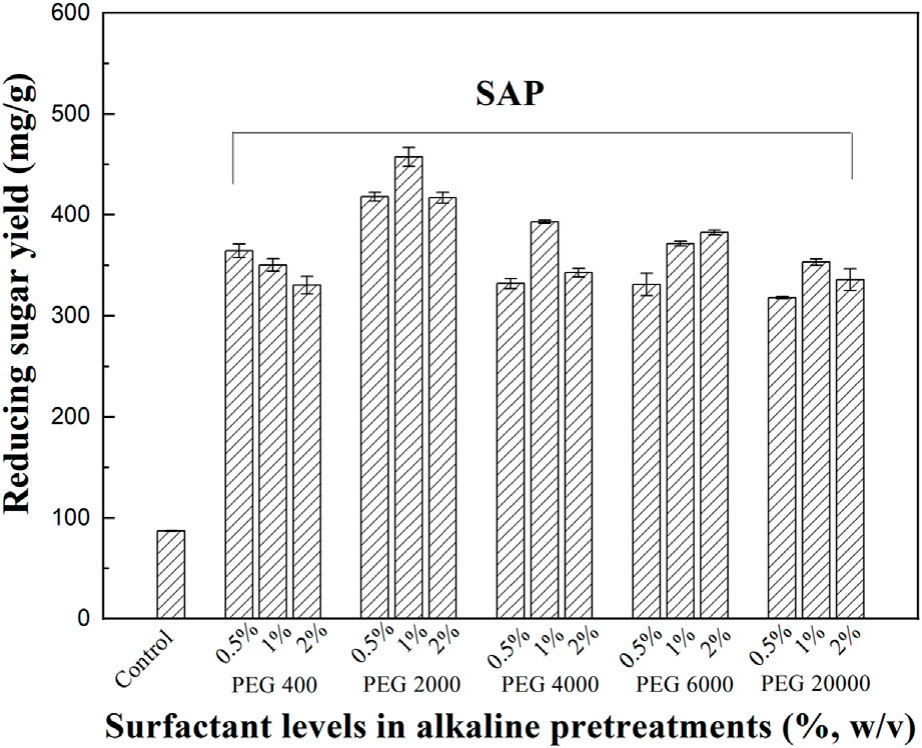
The effect of SAP with PEG surfactants on enzymatic hydrolysis.

Different surfactants including PEG 400, PEG 2000, PEG 4000, PEG 6000, PEG 20000, Tween 60, and CTAB were tested in SAP. The untreated and treated stalk samples were subjected to subsequent enzymatic hydrolysis and the results are shown in Figures 1, 2. The untreated sample of *M. sinensis*, which contains the original recalcitrant structure of the plant cell wall, had the lowest yield of reducing sugars after 72 h of the enzymatic hydrolysis. The digestibility of the pretreated stalks was significantly enhanced by AP using 0.6% NaOH alone.

The addition of the above surfactants enhanced the efficiency of the AP process. In cases of SAP assisted by 0.5% of Tween 60 and CTAB, obvious improvements in the enzymatic hydrolysis were recorded. As the surfactant concentrations in SAP became elevated (1 vs 0.5%), the corresponding sugar yields also increased. As shown in Figure 1, SAP assisted by 1% of Tween 60 improved the reducing sugar yields to 425.7 mg/g, which is 490% of that observed in the untreated sample. SAP assisted by PEG achieved a significant enhancement in the bioconversion of the pretreated samples. As shown in Figure 2, the addition of PEG 4000–6000 in SAP resulted in a good enhancement of the enzymatic hydrolysis of the pretreated samples. Among all tested surfactants (0.5–2%), SAP assisted by 1% of PEG 2000 yielded the highest reducing sugar yield (457.3 mg/g), which is 526% of that observed in the untreated sample.

Previous studies have reported that CTAB and PEG 6000 were most effective for enhancing enzymatic hydrolysis of pine fallen foliage (Pandey and Negi, 2015) in acid and alkaline pretreatments, respectively. Tween 80 and PEG 4000 assisted acid pretreatment of corn stalk indicated high sugar yields (Qing et al., 2010). In a recent study, the addition of sodium dodecyl benzene sulfonate (SDBS), which could reduce the surface tension, thereby increasing the diffusion of hydrogen ions and promoting the dissolution of hemicellulose and lignin, enhanced the hemicellulose removal from poplar wood chips during the mild acid hydrolysis (Wang et al., 2020). Enhanced sugar recovery from corn stalk was performed by a two-step pretreatment with Tween 80 and Ferric Nitrate to remove lignin and hemicellulose (Sun et al., 2019). A maximum sugar yield was reported when PEG 6000 was used for the pretreatment of chili post-harvest residue (Sindhu et al., 2018). In this study, SAP assisted by PEGs, such as PEG 2000, enhanced the sugar yields in the enzymatic hydrolysis, which may be resulted from enhanced structure modification and compositional disruption. Therefore, compositional analysis and microstructure observation of untreated and pretreated samples were carried out in the following studies.

### Compositional analysis of untreated and pretreated samples

**FIGURE 3:**
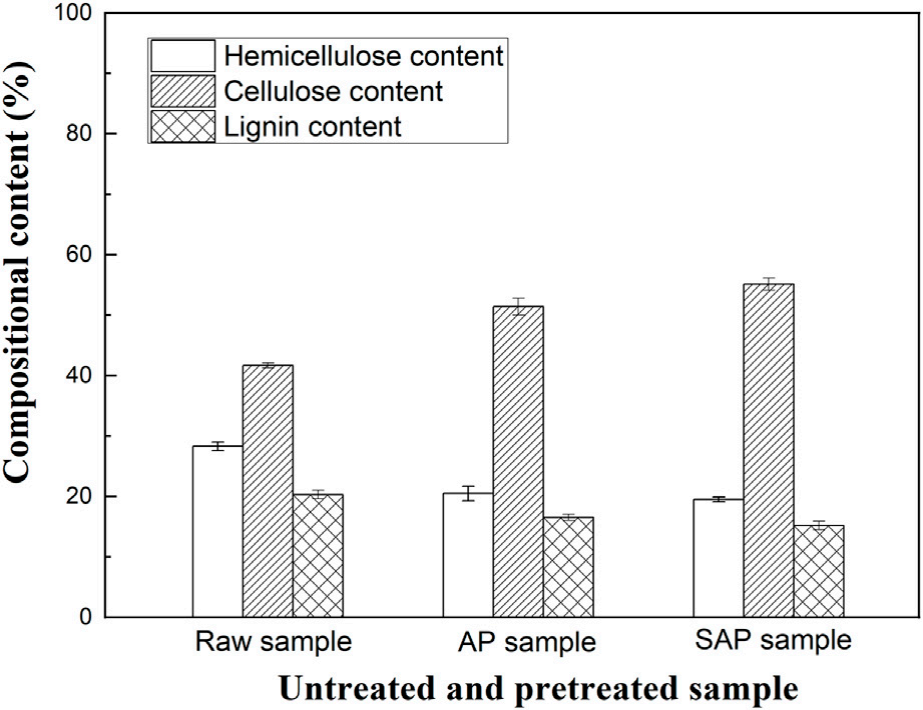
The compositional analysis of untreated and pretreated samples.

Results of the compositional analysis of the untreated and pretreated samples are shown in Figure 3. AP, which breaks the lignin structure by producing nucleophilic attacks, solubilizes lignin or hemicellulose from α-O-4 linkages (Shimizu et al., 2018). The present results showed that individual AP with 0.6% NaOH produced a significant delignification effect and hemicellulose removal (Figure 3). It should be noted that SAP with 1.0% PEG 2000 and 0.6% NaOH more significantly reduced the lignin and hemicellulose content of biomass to 15.2 and 19.5%, respectively, as compared to those achieved by individual AP (Figure 3). The corresponding cellulose content remarkably increased to 55.1%.

Different pretreatments decrease the recalcitrance of lignocellulosic wastes by reducing the lignin and hemicellulose components and exposing the cellulose to cellulases, thereby improving their bioconversion (Brienzo et al., 2017). The hemicellulose removal in the acid pretreatments, which increases the cellulose accessibility, improved the enzymatic hydrolysis efficiency of the pretreated samples (Tang et al., 2019). Moreover, a more significant delignification and higher reducing sugar recovery were observed in AP compared to acid pretreatments (Tang et al., 2019). AP, which can break or degrade some ester bonds and glycosidic linkages, could result in the reduction of the lignin–hemicellulose complex, cellulose swelling, and lignin removal, hence producing a considerable increase of bioconversion efficiency (Pandey and Negi, 2015; Wang et al., 2018). These results suggest that the enhanced lignin removal in SAP, which decreases the unproductive binding of cellulases to lignin, could play a vital role in producing a higher sugar release compared to AP (Figure 2).

### Microstructure observation of untreated and pretreated samples

**FIGURE 4:**
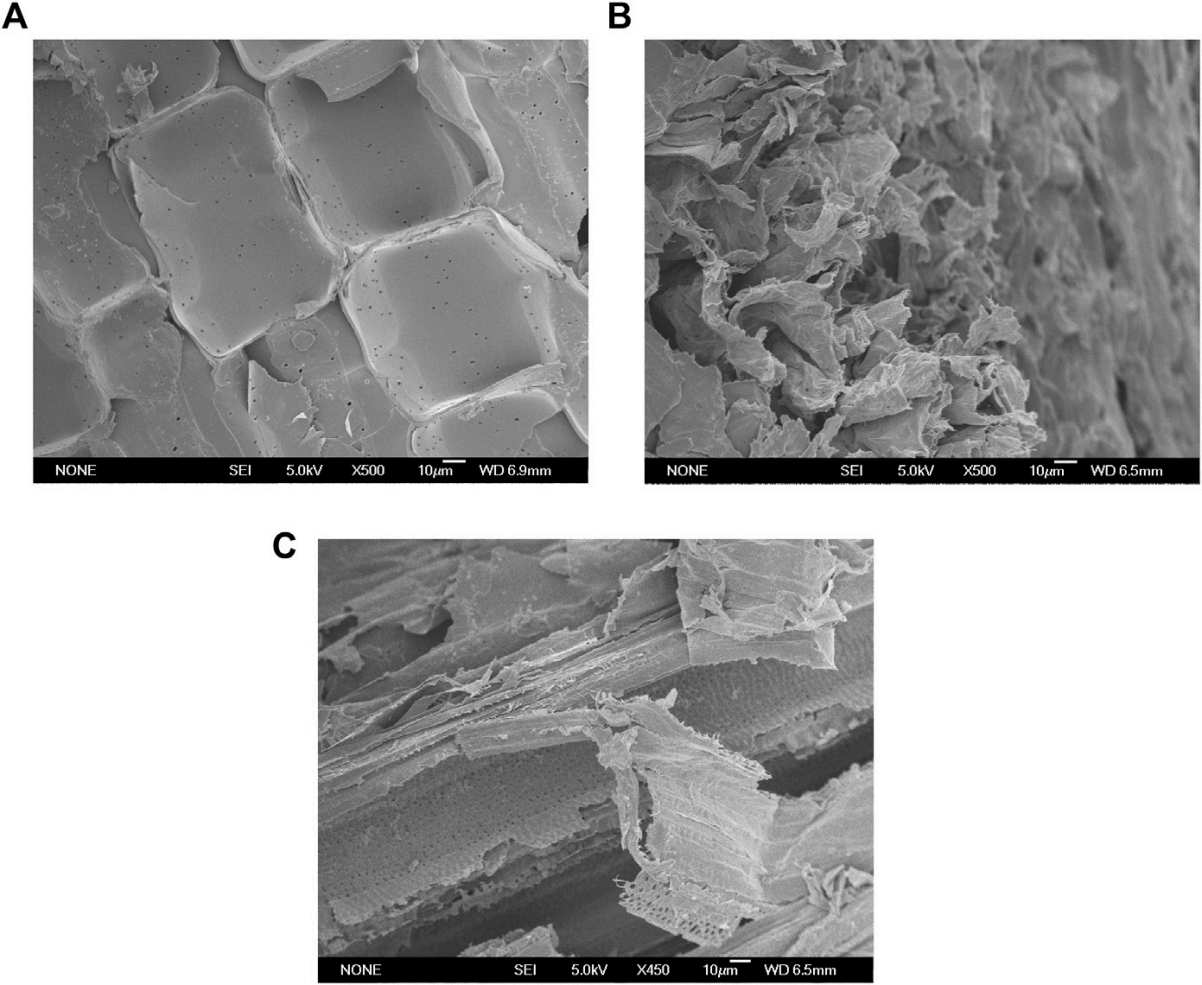
SEM observations of the stalk samples before and after pretreatments (500×). **(A)** Raw sample; **(B)** AP sample; **(C)** SAP sample.

The SEM at a magnification ratio of ×500 was used to observe morphological changes of the untreated and pretreated samples. As shown in Figure 4A, the untreated sample had a compact, rigid, and ordered structure, which could reduce the accessibility of cellulose to enzymes and microorganisms. A high degree of distortion was observed in the AP sample. As shown in Figure 4B, the cell wall surface of the AP sample was significantly disrupted, and the ordered boundaries of the cell wall became blurry. This could be due to the strong delignification and efficient removal of hemicellulose by AP. Compared to the untreated and AP sample, the cell wall microstructure changes of the SAP sample were more significant. As shown in Figure 4C, distorted surfaces and blurry boundaries were also observed in the SAP sample. Moreover, the surfactant addition in the SAP process may help alter the cell wall structures. Part of the surface layers seems to be remarkably disrupted and/or peeled off. Much more components, even those in the deep layers of cell walls, could be effectively dissolved. As a result, significant porous structures appeared on the surface and deep layers of the SAP sample (Figure 4A).

A strong disruption of the plant cell wall was also observed in the different biomass samples pretreated with acid/alkaline (Tang et al., 2019; Li W. C. et al., 2020). The hydrophilic and hydrophobic properties of surfactants (e.g., PEG and Tween) decrease the surface tension between liquid phases in the pretreatment stage, and help remove the hydrophobic compounds, such as lignin (Kataria et al., 2018; Sindhu et al., 2018). These results indicated that the surfactant addition further enhanced the alkali disruption of the cell walls and resulted in more cracks, fragments, porous structure, and lignin removal, hence producing much more reactive sites on the biomass surface. These changes increasingly help reduce the non-productive absorption of cellulases, increase the accessibility of SAP samples to cellulases, and consequently augment the subsequent enzymatic hydrolysis (Figures 2, 4). Recent studies have also confirmed that surfactant PEG 6000, sodium dodecyl sulphate (SDS) or Tween-mediated hydrothermal pretreatments obtained better delignification and hemicellulose removal, thereby enhancing the bioconversion of biomass wastes including Rye grass and chili post-harvest residue (Kataria et al., 2018; Sindhu et al., 2018; Xu et al., 2021).

### Effect of alkali concentrations in SAP on enzymatic hydrolysis

**FIGURE 5:**
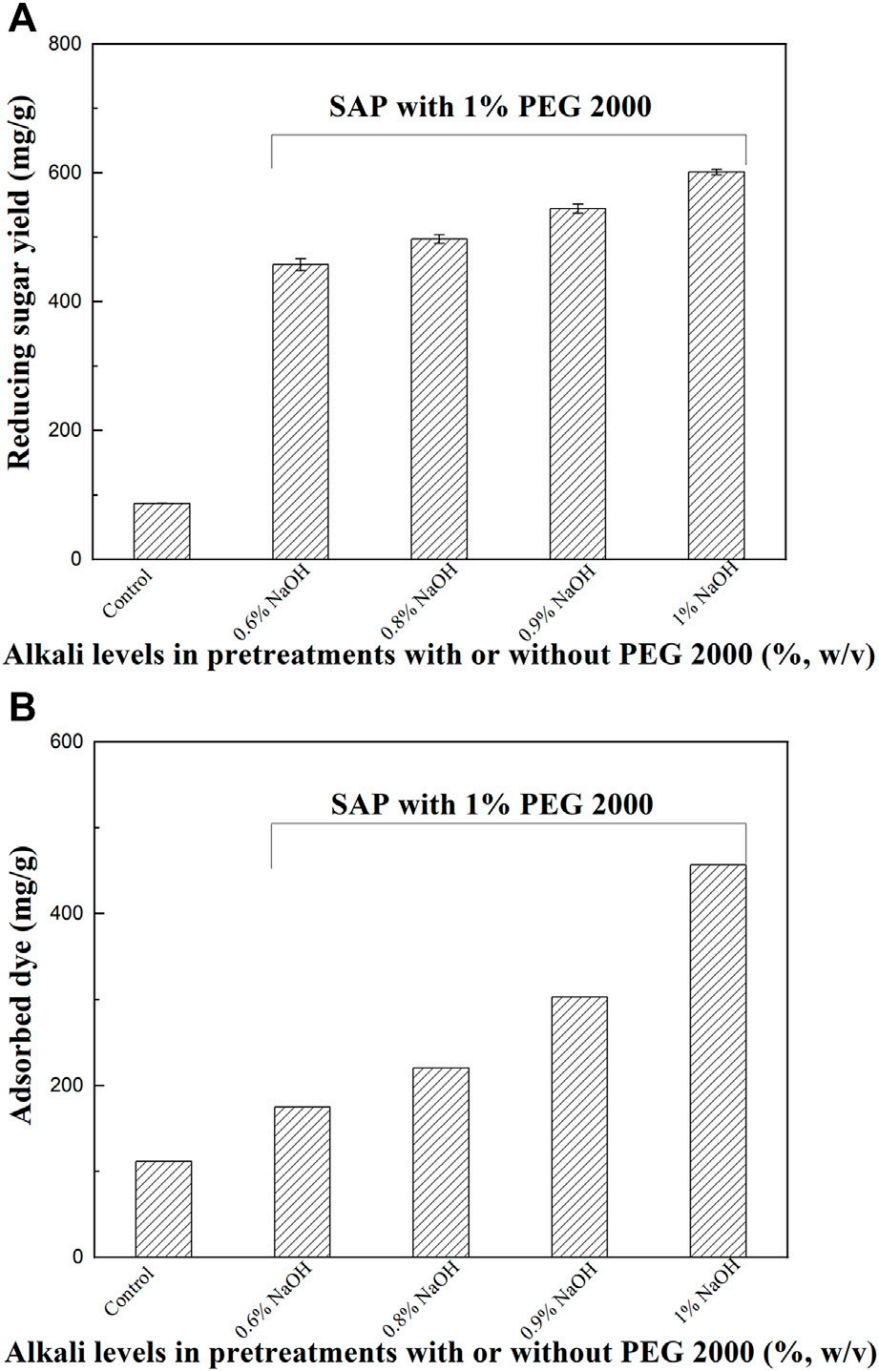
The effect of alkali concentrations in PEG 2000-mediated SAP on enzymatic hydrolysis **(A)** and accessibility **(B)** of these pretreated samples.

Interestingly, PEG 2000 assisted alkaline pretreatment, which help to produce better structure modification and/or extraction of dissolved lignin, enabled much more efficient saccharification compared to other tested surfactants; therefore, it was chosen for further studies. Effect of alkali concentrations of SAP assisted by PEG 2000 on enzymatic hydrolysis of the pretreated sample was investigated. Distinct differences in reducing sugar recovery were observed between the PEG 2000 assisted SAP and AP using alkali alone (Figure 1, Figure 5A). When 0.6–0.8% of NaOH solutions were used, the sugar yields of SAP samples in the enzymatic hydrolysis obtained an obvious increase compared to the raw sample. As NaOH concentrations in SAP were higher, the sugar yields also became higher. The SAP with 0.9–1.0% NaOH improved the reducing sugar yields to 544.4–601.2 mg/g, against 87.0 mg/g and 457.3 mg/g for the untreated and pretreated sample by SAP with 0.6% NaOH, respectively.

The surfactant addition in SAP significantly improved the alkali disruption of the cell walls and resulted in more cracks, porous structure, and lignin removal (Figure 4), which will help improve the accessibility of the pretreated stalks. As shown in Figure 5B, the adsorption capacity of the dye on raw sample was only 111.6 mg/g, while it reached 174.8 mg/g for SAP with 0.6% NaOH. As the NaOH concentrations in SAP were higher, the adsorption capacity of the dye on SAP samples also became higher (Figure 5B). For SAP with 1.0% NaOH, the absorbed dye reached the highest level of 456.6 mg/g, indicating a significantly improved cellulose accessibility of SAP sample to enzymes. As a result, the highest reducing sugar yield was also observed in this case.

### Effect of SEH on sugar yield

It is well known that the industrial application of bioethanol production is impeded by the high cost, in which the enzyme cost accounts for 20–30% (Tang et al., 2019). To further improve the hydrolysis efficiency and/or reduce the required enzyme loading ratios, surfactants including Tween 80, Tween 20, and PEG 2000, were used as representatives to evaluate the effect of the surfactant addition on enzymatic hydrolysis. In this SEH process, 8–15 FPU/g enzyme loading ratios were used.

**FIGURE 6:**
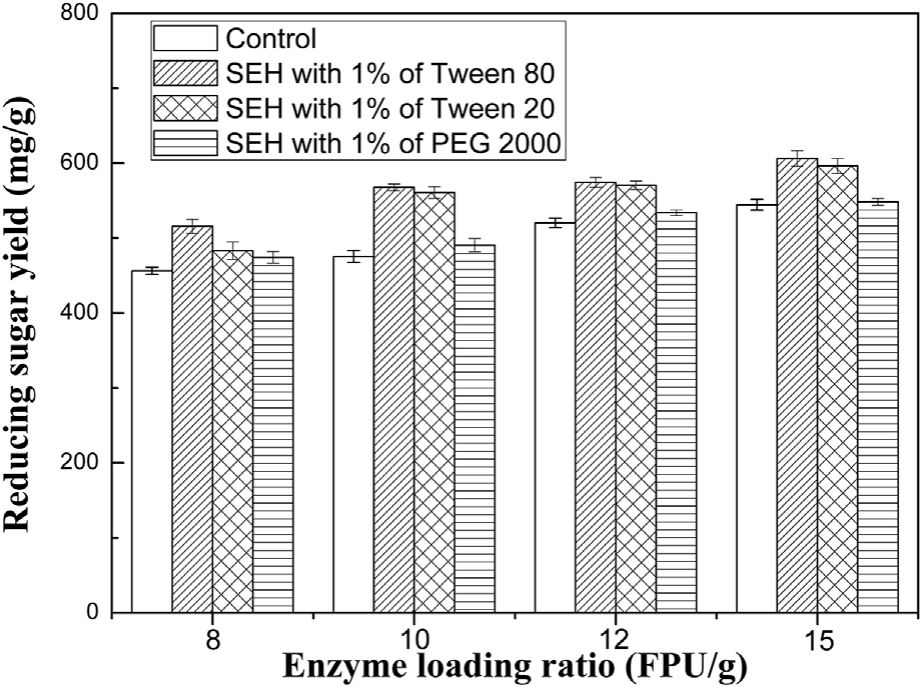
The effect of SEH of SAP sample on the fermentable sugar production.

As shown in Figure 6, the addition of PEG 2000 in the enzymatic hydrolysis of the SAP samples produced a slight increase (less than 4%) in the sugar yields, with 8–15 FPU/g enzyme loading ratios. Compared to PEG 2000, the addition of Tween 20 and Tween 80 achieved a higher enhancement of the hydrolysis efficiency, and surfactant addition was more effective in enhancing enzymatic hydrolysis with lower cellulase loading. For instance, the addition of 1% Tween 80 in the enzymatic hydrolysis using an enzyme loading of 8 FPU/g-10 FPU/g increased the sugar yield by 13.0–19.4% compared to the corresponding control, against the increase of 11.4% obtained for an enzyme loading of 15 FPU/g. In a previous study, the addition of Tween 80 with 0.25% concentration (w/v) also indicated an increase in the reducing sugar yields by about 50.5% for alkali-pretreated palm fruit bunch (Parnthong et al., 2017). The addition of 11.3% (w/v) of Tween 80 resulted in an improvement in cellulose digestibility by 36.2 and 7.8% for unwashed or washed biomass, respectively (Oladi and Aita, 2018). A maximum sugar yield of 445 mg/g was observed in the enzymatic hydrolysis of chili post-harvest residue pretreated by 3% PEG 6000 at 121 C for 60 min (Sindhu et al., 2018). In a recent study, the addition of 5 g/L of Tween 20, which could modify lignin surface properties and block lignin-cellulose interactions, improved the hydrolysis efficiency by 2.4–23.2% for acid-pretreated and alkali-pretreated substrates (Chen et al., 2018).

Moreover, the surfactant addition can either remarkably improve the sugar yields; or achieve comparable sugar yields with reduced enzyme loading. For instance, with the addition of 1% Tween 80, the enzymatic hydrolysis of the SAP sample obtained a significant increase (11.4%) in the sugar yield using the same enzyme loading (15 FPU/g); or using a much lower enzyme loading (10–12 FPU/g) for a small margin of increase (4.3–5.5%). Recent studies have reported that the addition of Tween 80 greatly shortens the hydrolysis time with a 50% reduction of enzyme loading to obtain the same level of glucose yield for pretreated sugarcane bagasse using FeCl_3_ and ethanol (Zhang et al., 2018). The mass balance in our previous studies revealed that AP significantly enhanced the enzymatic hydrolysis of pretreated substrates, thus producing a remarkable decrease in the amount of cellulase used (Li L. C. et al., 2020). Surprisingly, in the SEH process of the SAP sample, another substantial reduction of 33.3% in the enzyme loading was obtained to achieve even a higher sugar recovery (Table 1).

**TABLE 1:** Comparison of the fermentable sugar recovery from different biomass.

Biomass	Pretreatment conditions	Enzyme loading (FPU/g)	Sugar yield (mg/g)^1^	Ref
*Populus*	1% H_2_SO_4_, 120°C, 10 min	20	< 150 (GY)	Meng et al. (2015)
	1% NaOH, 120°C, 10 min	20	320 (GY)	
Wild rice grass	2% H_2_SO_4_, 121°C, 60 min	20	457	Sahoo et al. (2018)
*Eucalyptus*	12.5% [TBA][OH] or 2% NaOH, ultrasound irradiation	35	362.3–426.6	Li et al. (2016a)
*Pennisetum purpureum*	2% Ca(OH)_2_ or NaOH, 121°C, 60 min	15	324–537^2^	Phitsuwan et al. (2016)
Pine foliage	1% C-TAB, 1% H_2_SO_4_, 121°C, 60 min	100	588	Pandey and Negi, (2015)
	1% PEG-6000 1% NaOH, 121°C, 60 min	100	477	
Bamboo	1% NaOH, 3% Tween 80,121°C, 60 min	40	629	Li et al. (2016b)
Chili post-harvest residue	3% PEG 6000,121°C, 60 min	30	445	Sindhu et al. (2018)
*Miscanthus*	4% NaOH, 121°C, 20 min	--	590–700	Si et al. (2015)
	4% H_2_SO_4_, 121°C, 20 min	--	370–530	
*Miscanthus sinensis*	1.2% NaOH, 120°C, 30 min	15	526.5	Li et al. (2020b)
*Miscanthus sinensis*	0.9% NaOH, 1% PEG 2000,121 °C, 10 min	15	601.2	This study
*Miscanthus sinensis*	0.9% NaOH, 1% PEG 2000,121°C, 10 min	10–12	567.8–574.4	This study

Note: ^1^The soluble sugar yields were calculated based on per g pretreated stalk. ^2^The soluble sugar yields were calculated on the basis of the reported data of glucose and/or xylose yields in their studies. GY: glucose yield; Ref.: references.

The fermentable sugar yield from the *M. sinensis* sample pretreated by SAP was comparable to those observed in previous studies (Si et al., 2015; Tang et al., 2019; Li W. C. et al., 2020; Zoubiri et al., 2020). The maximum sugar levels of 150–629 mg/g were reported by acid or alkaline pretreatments of various lignocellulosic wastes, such as pine foliage, wild rice grass, bamboo, and *Eucalyptus* (Table 1). Total fermentable sugar yields of 370–700 mg/g have been obtained from *M. sinensis* biomass samples pretreated with 4% H_2_SO_4_ or 4% NaOH at 121°C for 20 min (Si et al., 2015; Li L. C. et al., 2020). In this study, the fermentative sugar yield observed so far was more than 567.8 mg/g-574.4 mg/g of *M. sinensis* pretreated by SAP, with a much lower enzyme loading of 10–12 FPU/g, thus indicating a promising strategy for efficient bioenergy production from *M. sinensis* (Table 1).

## Conclusion

The impact of surfactants on alkaline pretreatment and enzymatic hydrolysis of *M. sinensis* was investigated. The SAP using PEG 2000, which produced more efficient removal of lignin and hemicellulose from the pretreated sample as well as stronger disruption of microstructure, significantly improved the fermentable sugar production from *M. sinensis*. Moreover, the addition of Tween 80 in the enzymatic hydrolysis (i.e., SEH) of the SAP sample, which could modify lignin surface properties and block lignin-cellulose interactions, achieved a higher sugar recovery, even with a substantially reduced cellulase loading. These results indicate that the strategy of coupling SAP with SEH indeed has the potential to achieve the effective bioconversion of *M. sinensis*, which makes this material a promising candidate for bioenergy production.

## Data Availability

The original contributions presented in the study are included in the article/Supplementary Material, further inquiries can be directed to the corresponding author.
